# Testing the limits of contextual constraint: Interactions with word frequency and parafoveal preview during fluent reading

**DOI:** 10.1080/17470218.2017.1327981

**Published:** 2018-01-01

**Authors:** Sara C Sereno, Christopher J Hand, Aisha Shahid, Bo Yao, Patrick J O’Donnell

**Affiliations:** 1 Institute of Neuroscience and Psychology, University of Glasgow, Glasgow, UK; 2 School of Psychology, University of Glasgow, Glasgow, UK; 3 School of Health and Life Sciences, Glasgow Caledonian University, Glasgow, UK; 4 Division of Neuroscience and Experimental Psychology, University of Manchester, Manchester, UK; * We are deeply saddened to report that Patrick J. O’Donnell passed away in April 2016.

**Keywords:** Contextual predictability, word frequency, parafoveal preview, eye movements, reading

## Abstract

Contextual constraint is a key factor affecting a word’s fixation duration and its likelihood of being fixated during reading. Previous research has generally demonstrated additive effects of predictability and frequency in fixation times. Studies examining the role of parafoveal preview have shown that greater preview benefit is obtained from more predictable and higher frequency words versus less predictable and lower frequency words. In two experiments, we investigated effects of target word predictability, frequency and parafoveal preview. A 3 (Predictability: low, medium, high) × 2 (Frequency: low, high) design was used with Preview (valid, invalid) manipulated between experiments. With valid previews, we found main effects of Predictability and Frequency in both fixation time and fixation probability measures, including an interaction in early fixation measures. With invalid preview, we again found main effects of Predictability and Frequency in fixation times, but no evidence of an interaction. Fixation probability showed a weak Predictability effect and Predictability–Frequency interaction. Predictability interacted with Preview in early fixation time and fixation probability measures. Our findings suggest that high levels of contextual constraint exert an early influence during lexical processing in reading. Results are discussed in terms of models of language processing and eye movement control.

After controlling for the effects of word length (e.g., Rayner, Sereno, & Raney, 1996), the two key factors affecting fixation time on a word in reading are its predictability from the prior context and its frequency of occurrence (e.g., Rayner, 1998, 2009). Numerous studies examining Predictability have demonstrated that high predictable (HP) words are fixated for shorter durations and less often than low predictable (LP) words (e.g., Balota, Pollatsek, & Rayner, 1985; Ehrlich & Rayner, 1981; Hand, Miellet, O’Donnell, & Sereno, 2010; Hand, O’Donnell, & Sereno, 2012; Kennedy, Pynte, Murray, & Paul, 2013; Kliegl, Grabner, Rolfs, & Engbert, 2004; McDonald & Shillcock, 2003; Miellet, Sparrow, & Sereno, 2007; Morris, 1994; Rayner, Ashby, Pollatsek, & Reichle, 2004; Rayner & Well, 1996). Similarly, studies examining Frequency have consistently demonstrated that high frequency (HF) words are fixated for shorter durations and less often than low frequency (LF) words (e.g., Hand et al., 2010; Hand et al., 2012; Inhoff & Rayner, 1986; Just & Carpenter, 1980; Kennedy et al., 2013; Kliegl et al., 2004; Miellet et al., 2007; Rayner & Duffy, 1986; Rayner et al., 1996; Scott, O’Donnell, & Sereno, 2012; Sereno, 1992; Sereno, O’Donnell, & Rayner, 2006; Sereno, Pacht, & Rayner, 1992; Sereno & Rayner, 2000a; Slattery, Pollatsek, & Rayner, 2007).

Eye movement studies which have explicitly investigated the combined effects of Predictability and Frequency in reading, in comparison, are far less prevalent (e.g., Hand et al., 2010; Hand et al., 2012; Kliegl et al., 2004; Miellet et al., 2007; Rayner, Ashby, et al., 2004). Such studies have typically reported additive effects of Predictability and Frequency in fixation time measures. A separate set of studies, however, has found interactive effects of Predictability and Frequency, with greater contextual facilitation for LF than HF words (for a discussion, see Hand et al., 2010). These studies have used word naming or lexical decision paradigms (e.g., Stanovich & West, 1983; West & Stanovich, 1982), event-related potentials (ERPs; for example, Sereno, Brewer, & O’Donnell, 2003; Van Petten & Kutas, 1990) and eye fixation times (e.g., Inhoff, 1984). To address these discrepancies and to examine the role of parafoveal preview (i.e., information acquired parafoveally from a target word, from the prior fixation), Hand et al. (2010) manipulated Predictability and Frequency but additionally employed a *post hoc* technique linked to launch distance (i.e., the distance from the pretarget fixation to the target). Based on the fact that visual acuity drops off as a function of retinal eccentricity (e.g., Miellet, O’Donnell, & Sereno, 2009), their approach assumes that the degree of parafoveal information acquired about a target is negatively correlated with launch distance. They used Preview levels (i.e., launch distance to target) of Near, Middle and Far (1-3, 4-6 and 7-9 characters away, respectively) and found interactive Predictability–Frequency findings for both Near and Middle distances. The opposing nature of these interactions – specifically showing reliably greater Predictability effects for LF words at Near distances, but for HF words at Middle distances – resulted in an overall additive pattern of Predictability and Frequency.^1^

Although there are merits to Hand et al.’s (2010) approach, eye movement studies have typically manipulated the quality of the reader’s parafoveal preview of a target word by changing the appearance of that target before it is directly fixated. In the boundary technique, for example, readers parafoveally view either the valid target word or an invalid letter string which changes to the target when their eyes cross a prespecified invisible boundary (Rayner, 1975). The extent of parafoveal analysis of a target can then be indexed by the relative processing advantage of valid versus invalid previews. Using the boundary technique, it has been demonstrated that both Predictability and Frequency effects are modulated by Preview. Specifically, readers extract more information from parafoveal words that are HP compared with LP (e.g., Balota et al., 1985; Drieghe, Rayner, & Pollatsek, 2005) and from ones that are HF compared with LF (e.g., Inhoff & Rayner, 1986; Reingold, Reichle, Glaholt, & Sheridan, 2012).

The nature of the Predictability–Frequency interaction – whether it is additive or interactive – has implications for models of language processing. For example, an interactive account posits that context can directly influence lexical access (e.g., McClelland, 1987), whereas a modular account holds that context can only operate on the output of the lexical processor (e.g., Fodor, 1983). Accordingly, determining the temporal locus of contextual effects – whether they occur earlier or later, during lexical or post-lexical processing – is a key issue in understanding the circuitry of word recognition. One approach that has been used to assess the relative timing of different processes is additive factors (e.g., Sternberg, 1969). Word frequency effects are considered to index lexical access (e.g., Balota, 1990; Sereno & Rayner, 2000b, 2003; Sereno, Rayner, & Posner, 1998). An interaction of predictability with word frequency would suggest these variables are concurrently processed, indicating an early, lexical locus of context effects. Conversely, an additive pattern of effects would suggest that contextual processing is relatively delayed, occurring postlexically.

One concern of prior Predictability–Frequency studies is related to the relative strength of the biasing contexts that have been used, operationalized in terms of a target word’s Cloze value (i.e., the probability that the target is correctly guessed given its preceding context). The average Cloze values for items categorized as ‘HP’ in past eye movement studies typically vary between 0.50 and 0.70, below the top end of the scale. One study that employed genuine HP targets was that of Rayner and Well (1996). They defined three levels of contextual constraint based on Cloze probabilities: low (0.04, range: 0.03-0.08), medium (0.41, range: 0.13-0.68) and high (0.86, range: 0.73-1.00). They found longer fixations on low compared with medium or high constraint targets. Although fixation times on medium and high constraint targets did not differ, Rayner and Well’s targets were HF words. The Predictability–Frequency interactions reported in past studies, however, arise from greater contextual effects in LF than in HF words.

The current study employed two experiments to investigate the nature of Predictability–Frequency effects on eye movement behavior during reading, when Preview of the parafoveal target was valid (Experiment 1) or invalid (Experiment 2). Several aspects of our approach are worth noting. First, we investigated low, medium, as well as genuinely high levels of Predictability (LP, MP and HP), with targets having average Cloze values of 0.01, 0.54 and 0.96, respectively. Second, target words were embedded in two-sentence passages of text. The first sentence comprised the main context; the second was relatively neutral and contained the target word. The majority of past eye movement studies investigating Predictability have utilized single-line sentences, with context limited to the first few words. A greater amount of content preceding a target may allow context effects to develop more fully (e.g., Hand et al., 2010). Third, individual contexts were devised for each target word. Past studies have often used the same sentential frame to accommodate two different targets (participants only see one version), thereby generating items in two conditions (e.g., HF vs LF targets of a certain predictability; HP vs LP targets of a certain frequency). While the text preceding the target is identical across conditions, this quality of the stimuli is realized in different participants. Finally, the invalid parafoveal previews were pronounceable nonwords retaining the same overall word shape as their eventual targets (i.e., in terms of ascending, descending and in-line characters), but without positionally overlapping letters (e.g., *phem* for *glue; torm* for *hair*). Sereno and Rayner (2000a) suggested that previews that are pronounceable and relatively regular in terms of their orthography are less likely to attract parafoveal awareness of their complexity and subsequent costs in foveal processing (see also Reingold et al., 2012).

The pattern of our results will address the degree to which contextual factors can influence lexical processing. Although prior reading studies have generally reported additive effects of Predictability and Frequency, their ‘HP’ conditions do not provide maximal contextual bias for targets. Moreover, while it is recognized that parafoveal preview plays a key role in the acquisition of information related to an upcoming target word’s predictability or its frequency, preview effects related to both factors in combination are less well understood.

## Experiment 1

Experiment 1 investigated the joint effects of Predictability (LP, MP and HP) and Frequency (LF and HF) in normal reading (i.e., with valid parafoveal previews).

### Method

#### Participants

Forty native English-speaking members of the University of Glasgow community (28 females; mean age: 23) took part in the experiment. They all had normal or corrected-to-normal vision, had not been diagnosed with any reading disorder, and were either paid £6 or given course credit for their participation. The study conformed to British Psychological Society ethical guidelines and protocols.

#### Materials and design

Passages comprised two single-line sentences, with each sentence limited to 70 character spaces. The first sentence was more or less biasing towards the upcoming target. Targets appeared in the second sentence and were positioned near the middle of the line (reducing the possibility of sentence-initial or sentence-final fixations on the target). Care was taken to ensure that the pretarget region of the second sentence was relatively neutral and did not contain, for example, intralexical primes of the subsequent target. That is, while the inclusion of associative or semantic primes that proximally precede targets (e.g., *buttered popcorn; bride and groom; a sheet of paper; baked a cake*) facilitates target identification, such ‘context’ is considered to originate at the lexical rather than discourse or message level (e.g., Forster, 1979). Care was also taken to ensure that, in cases of low predictability, the target was not semantically anomalous but was merely a word that was far less expected. When anomalous constructions are used (e.g., *inflate the carrot* vs *chop the carrot*), an immediate disruptive effect on eye movements is observed (e.g., Rayner, Warren, Juhasz, & Liversedge, 2004).

To determine target word predictability, a superset of 200 items was presented in separate norming tasks – a Cloze task and a predictability rating task – to two different groups of participants, neither of whom participated in the main experiments. In the Cloze task, 20 participants were given each item up to, but not including, the target and were required to generate the next word of the passage. Items were scored as ‘1’ for correct responses and ‘0’ for all other guesses. In the rating task, 20 additional participants read all items in their entirety, with target words printed in bold font. Participants indicated how predictable they considered the target word to be on a scale of 1 (*highly unpredictable*) to 7 (*highly predictable*). Experimental items were selected and matched across conditions based on their Cloze probabilities and ratings, as well as their frequency and length. Word frequencies were acquired from the British National Corpus (BNC), a database of 90 million written word tokens (Davies, 2004). Word length was limited to a range of four to eight letters.

A 3 (Predictability: LP, MP, HP) × 2 (Frequency: LF, HF) repeated-measures design was used. There were 25 items in each of the six conditions. All target words are listed in Table 1. Target word specifications of length, frequency, Cloze and predictability rating values are presented in Table 2. Table 3 provides example materials across conditions.

**Table 1.** Target words across experimental conditions.

LF			HF		
LP	MP	HP	LP	MP	HP
peas	lust	glue	bear	seat	hair
bark	chef	kite	goal	text	door
dusk	cult	plug	ball	food	city
drum	cape	cage	land	week	body
jail	sofa	oven	room	life	view
sewer	gravy	zebra	phone	plant	dream
melon	spade	camel	voice	price	stage
attic	shark	stain	power	light	class
stall	tooth	witch	party	staff	court
icing	feast	towel	house	night	money
parrot	poster	bonnet	memory	volume	breath
grease	candle	tailor	career	prison	labor
puzzle	hammer	heater	garden	winter	window
pillow	ballet	fridge	member	leader	police
pepper	hunter	spider	health	friend	market
rabbit	collar	poison	public	street	family
scooter	sunburn	lobster	factory	patient	station
blender	shampoo	malaria	respect	disease	address
reunion	cushion	laundry	weekend	pattern	library
balloon	posture	perfume	culture	village	husband
refugee	drought	costume	picture	meeting	morning
pottery	cleaner	receipt	history	country	council
ornament	necklace	confetti	mountain	religion	painting
diabetes	inventor	lipstick	daughter	football	magazine
civilian	burglary	equality	property	security	hospital

LF, low frequency; HF, high frequency; LP, low predictability; MP, medium predictability; HP, high predictability.

**Table 2.** Target word specifications across experimental conditions.

	LF			HF		
	LP	MP	HP	LP	MP	HP
Length						
Mean	5.88	5.88	5.88	5.88	5.88	5.88
*SD*	1.33	1.33	1.33	1.33	1.33	1.33
Min	4.00	4.00	4.00	4.00	4.00	4.00
Max	8.00	8.00	8.00	8.00	8.00	8.00
Frequency						
Mean	6.98	7.26	6.67	179.57	178.95	179.86
*SD*	3.88	4.01	3.58	141.99	133.78	109.79
Min	0.62	0.67	0.71	43.00	48.76	45.59
Max	14.90	14.62	16.22	547.72	611.76	363.53
Cloze						
Mean	0.01	0.52	0.96	0.01	0.56	0.97
*SD*	0.02	0.17	0.05	0.02	0.16	0.04
Min	0.00	0.20	0.85	0.00	0.20	0.90
Max	0.05	0.75	1.00	0.05	0.75	1.00
Predictability rating						
Mean	4.28	5.58	6.21	4.47	5.81	6.26
*SD*	0.66	0.69	0.34	0.74	0.71	0.24
Min	3.17	3.72	5.50	3.50	3.78	5.83
Max	5.61	6.44	6.72	5.94	6.56	6.72

LF, low frequency; HF, high frequency; LP, low predictability; MP, medium predictability; HP, high predictability. Condition means are shown with standard deviations (*SD*s) and item minimum (min) and item maximum (max) values. Units of measurement are as follows: length in number of letters; frequency in occurrences per million; Cloze probability on a scale of 0 (*target never guessed*) to 1 (*target always guessed*); and predictability rating on a scale of 1 (*highly unpredictable*) to 7 (*highly predictable*).

**Table 3.** Example materials.

Condition	Passages comprised of context and target sentences
LF-LP	Anna always remembers to collect her morning paper on the way to work. She enjoys the *puzzle* pages and eagerly tries to finish the crossword.
LF-MP	Dave wanted to build a new bookcase but couldn’t find his toolbox. Eventually, he had to borrow a *hammer* and nails from his neighbour.
LF-HP	At work, the boiler had broken and we were freezing at our desks. We arranged for a portable *heater* to be brought into the office.
HF-LP	Local businesses donated to a regeneration fund for the town centre. There were plans for a *garden* to be built with colourful flowers.
HF-MP	Many animals must hibernate in order to survive harsh climates. At the end of the *winter* they will wake up and forage for food.
HF-HP	The young boy recklessly kicked his ball in front of the house. One day, he broke a *window* and blamed it on his little brother.

LF, low frequency; HF, high frequency; LP, low predictability; MP, medium predictability; HP, high predictability. Target words are italicized.

Note that passages were displayed to participants as two single-line sentences.

Finally, as we did not explicitly control the length of the word before the target, a *post hoc* examination of our materials considered whether there were any systematic differences in pretarget word length across conditions. Such differences could potentially lead to different levels of parafoveal preview.^2^ Mean pretarget word lengths were 3.08, 3.24, 3.52, 2.96, 3.44 and 3.60 characters for LF-LP, LF-MP, LF-HP, HF-LP, HF-MP and HF-HP conditions, respectively. A two-way analysis of variance (ANOVA) by items revealed no differences in pretarget word length based on target Predictability (*F_2_*(2, 48) =1.04, *p*>0.35), Frequency (F_2_<1) or their interaction (F_2_<1).

#### Apparatus

Eye movements were monitored via an SR Research Desktop-Mount EyeLink 2K eyetracker (spatial resolution: 0.01°), with participants’ heads stabilized via a chin/forehead rest. Viewing was binocular and eye position was sampled from the right eye at 1000 Hz. EyeTrack software (http://www.psych.umass.edu/eyelab/software/) was used to control text presentation. Text (black letters on a white background, 14-point nonproportional Bitstream Vera Sans Mono font, quadruple line spacing) was presented on a Dell P1130 19″ flat screen cathode ray tube (CRT; 1024 × 768 resolution; 150 Hz refresh rate). At a viewing distance of 72 cm, approximately four characters of text subtended 1° of visual angle.

#### Procedure

Participants were instructed to read normally for comprehension and that questions would appear after some of the trials to ensure they were paying attention. After an initial calibration (9-point, full horizontal and vertical range of display) and validation procedure, participants read three practice passages before reading the 150 experimental passages (order randomized). Participants self-paced their breaks, and calibration and validation procedures were repeated after each break and as necessary throughout the experimental session. Yes–No comprehension questions followed one-third of the trials; on average, participants answered 98% correct.

### Results

To prepare the eyetracking data for statistical analyses, a suite of data management programs (e.g., EyeDoctor, EyeDry; http://www.psych.umass.edu/eyelab/software/) were used. The target region comprised the target word and the space before it. Lower and upper cutoff values for individual fixations were 100 and 650 ms, respectively. Data were additionally eliminated if there was a blink or track loss on the target, or if a first-pass fixation on the target was either the first or last fixation on that line. Overall, 5% of the data were excluded for these reasons. The percentages of the remaining data for single fixation, immediate refixation and first-pass skipping of the target were 69%, 4% and 27%, respectively.

**Figure 1.**
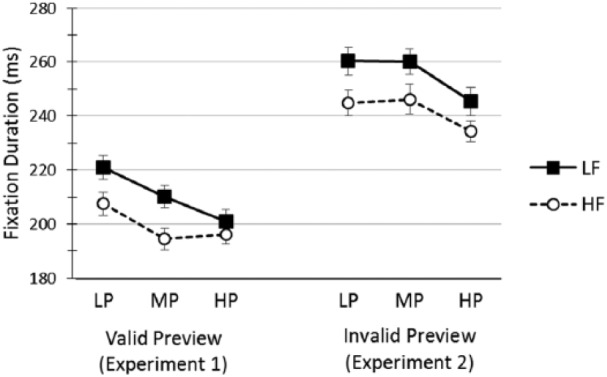
Single fixation duration (ms) on target words (with standard error bars) as a function of Predictability (LP, MP, HP), Frequency (LF, HF) and Preview (Valid, Invalid). LF, low frequency; HF, high frequency; LP, low predictability; MP, medium predictability; HP, high predictability.

The resulting data were analyzed over a number of standard measures: first fixation duration (FFD; the initial first-pass fixation duration, regardless of whether the target was refixated); single fixation duration (SFD; first-pass fixation time when a target was only fixated once); gaze duration (GD; the sum of all first-pass fixations before the eyes move to another word); total fixation time (TT; the sum of all fixations, including regressions or second-pass fixations); and the probability of making a first-pass fixation on the target (PrF; note that this is calculated as a proportion of valid trials only). We additionally examined the first-pass reading time of the first sentence (Sent1) of each passage, expressed in milliseconds per character (ms/char). This was done to confirm that any effects observed across conditions could not be attributed to variations in Sent1 length or reading speed across stimuli. All analyses of Sent1 data yielded nonsignificant effects (all *F*s < 1) and will not be discussed further. The average values across all measures (with standard deviations) are presented in Table 4. As the majority of first-pass target word fixations were single fixations, the SFD means across conditions are displayed in Figure 1.

**Table 4.** Means (standard deviations) of fixation measures across conditions in Experiments 1 and 2.

	LF			HF		
	LP	MP	HP	LP	MP	HP
Experiment 1						
FFD	219 (28)	210 (27)	200 (26)	207 (26)	195 (26)	196 (23)
SFD	221 (28)	210 (27)	201 (27)	208 (27)	194 (25)	196 (23)
GD	234 (32)	219 (31)	213 (36)	217 (29)	201 (27)	200 (25)
TT	252 (40)	240 (41)	227 (40)	237 (34)	219 (37)	209 (31)
PrF	0.78 (0.13)	0.75 (0.15)	0.73 (0.15)	0.74 (0.13)	0.70 (0.14)	0.70 (0.16)
Sent1	29 (5)	30 (5)	30 (4)	30 (4)	30 (5)	30 (4)
Experiment 2						
FFD	252 (31)	254 (28)	237 (31)	243 (32)	241 (33)	232 (26)
SFD	260 (34)	260 (29)	245 (33)	244 (30)	246 (35)	234 (24)
GD	295 (49)	285 (46)	280 (74)	273 (47)	274 (47)	257 (45)
TT	338 (96)	327 (112)	318 (128)	318 (96)	311 (101)	290 (85)
PrF	0.84 (0.11)	0.90 (0.09)	0.88 (0.08)	0.86 (0.09)	0.84 (0.10)	0.88 (0.08)
Sent1	30 (4)	30 (5)	30 (4)	30 (5)	30 (5)	30 (4)

LF, low frequency; HF, high frequency; LP, low predictability; MP, medium predictability; HP, high predictability; FFD, first fixation duration; SFD, single fixation duration; GD, gaze duration; TT, total fixation time; PrF, probability of fixation; Sent1, reading time on first sentence. For reading time measures, mean values are in milliseconds for FFD, SFD, GD and TT and milliseconds per character for Sent1.

For all measures, 3 (Predictability: LP, MP, HP) × 2 (Frequency: LF, HF) ANOVAs were conducted both by participants (*F*_1_) and by items (*F*_2_) and are reported in Table 5. Follow-up contrasts for Predictability are presented in Table 6. These analyses are appropriate for our data and are comparable with prior studies (e.g., Hand et al., 2010; Rayner, Ashby, et al., 2004).

**Table 5.** Main effects and interactions by participants (*F*_1_) and by items (*F*_2_) across measures in Experiment 1.

	Predictability			Frequency			Predictability × Frequency		
	*F*	*MSE*	*p*	*F*	*MSE*	*p*	*F*	*MSE*	*p*
FFD									
*F*_1_	22.21	212	<0.001	38.34	184	<0.001	3.95	158	<0.05
*F*_2_	13.14	198	<0.001	20.18	191	<0.001	2.93	202	0.063
SFD									
*F*_1_	24.69	216	<0.001	39.89	193	<0.001	3.96	165	<0.05
*F*_2_	16.67	178	<0.001	21.15	215	<0.001	3.57	217	<0.05
GD									
*F*_1_	27.29	291	<0.001	38.62	384	<0.001	<1		
*F*_2_	13.62	329	<0.001	18.63	449	<0.001	<1		
TT									
*F*_1_	21.11	698	<0.001	28.34	658	<0.001	<1		
*F*_2_	13.69	626	<0.001	10.18	900	<0.01	<1		
PrF									
*F*_1_	4.22	0.009	<0.05	11.69	0.010	<0.01	<1		
*F*_2_	4.22	0.006	<0.05	10.28	0.007	<0.01	<1		

MSE, mean squared error; FFD, first fixation duration; SFD, single fixation duration; GD, gaze duration; TT, total fixation time; PrF, probability of fixation. Degrees of freedom are as follows: *F*_1_(2, 78) and *F*_2_(2, 48) for Predictability; *F*_1_(1, 39) and *F*_2_(1, 24) for Frequency; and *F*_1_(2, 78) and *F*_2_(2, 48) for Predictability × Frequency.

**Table 6.** Predictability contrasts by participants (*p*_1_) and by items (*p*_2_) across measures in Experiment 1.

	Mean			LP vs MP		LP vs HP		MP vs HP	
	LP	MP	HP	*p*_1_	*p*_2_	*p*_1_	*p*_2_	*p*_1_	*p*_2_
FFD	213	202	198	<0.001	<0.001	<0.001	<0.001	0.074	>0.25
SFD	214	202	199	<0.001	<0.001	<0.001	<0.001	0.116	>0.15
GD	225	210	206	<0.001	<0.001	<0.001	<0.001	>0.15	>0.45
TT	245	230	218	<0.001	<0.01	<0.001	<0.001	<0.01	<0.05
PrF	0.76	0.73	0.72	0.050	<0.05	<0.01	<0.01	>0.40	>0.40

LP, low predictability; MP, medium predictability; HP, high predictability; FFD, first fixation duration; SFD, single fixation duration; GD, gaze duration; TT, total fixation time; PrF, probability of fixation. Mean values are in milliseconds for FFD, SFD, GD and TT.

Across all target word processing measures, both main effects of Predictability and Frequency were significant (see Table 5). For Predictability, follow-up contrasts showed that fixation durations were significantly longer for LP than for both MP and HP targets (see Table 6). With the exception of TT by participants, follow-up contrasts comparing fixation times on MP versus HP targets were not significant (see Table 6). Unlike fixation duration measures, the PrF difference between LP and MP targets was only marginally significant by subjects (see Table 6). The pattern of PrF effects for LP-HP (significant) and MP-HP (not significant) contrasts, however, was similar to that for fixation time measures (see Table 6). For Frequency, fixation times on LF words were longer than those on HF words (FFD: 210 vs 199 ms, SFD: 211 vs 199 ms, GD: 222 vs 206 ms and TT: 240 vs 222 ms, respectively) and participants were more likely to fixate LF than HF targets (0.76 vs 0.72).

A significant Predictability × Frequency interaction was observed in early measures of target word processing, namely, in FFD and SFD (see Table 5 and Figure 1). Predictability contrasts for LF words (LP vs MP vs HP) were all significant (all *F*_1_s > 10.05, *p*_1_s < 0.01; all *F*_2_s > 5.40, *p*_2_s < 0.05). However, for HF words, such contrasts only reached significance in LP-MP and LP-HP comparisons (all *F*_1_s > 14.65, *p*_1_s < 0.001; all *F*_2_s > 4.15, *p*_2_s < 0.05). The HF-MP and HF-HP conditions did not differ (all *F*s < 1). For Frequency contrasts (LF vs HF), significant effects were found in LP and MP conditions (all *F*_1_s > 20.80, *p*_1_s < 0.001; all *F*_2_s > 10.80, *p*_2_s < 0.01), but not in the HP condition (FFD: *F*_1_ = 2.62, *p*_1_ = 0.110; SFD: *F*_1_ = 2.87, *p*_1_ = 0.094; all *F*_2_s < 1). No evidence of an interaction was found in the remaining measures of GD, TT or PrF (see Table 5).

### Discussion

Unlike previous investigations (e.g., Hand et al., 2010; Rayner, Ashby, et al., 2004), we observed a significant interaction between Predictability and Frequency in early measures of lexical processing. Specifically, in the HP condition, there was no reliable difference in processing times associated with LF and HF words. Therefore, it seems that a strongly biasing context can neutralize word frequency effects, acting within a developing discourse to favorably constrain the set of candidate words.

## Experiment 2

Experiment 2 investigated the Predictability–Frequency interaction under conditions of invalid parafoveal preview. Although reading with invalid previews slows fixation times (e.g., Blanchard, Pollatsek, & Rayner, 1989; Sereno & Rayner, 2000a), it provides an opportunity to gauge the type and amount of information that is acquired from the target before its fixation.^3^ To reduce potentially disruptive effects of a false parafoveal stimulus, we employed orthographically legal, pronounceable nonword invalid previews whose overall word shape was similar to that of the target.

### Method

#### Participants

Forty participants (29 females, mean age: 22) having the same characteristics as described in Experiment 1 took part in the experiment and received similar compensation. None had participated in Experiment 1 or in the Cloze or rating tasks.

#### Materials and design

Experiment 2 used the same stimuli as Experiment 1 with one exception. A boundary paradigm (e.g., Rayner, 1975) was employed to present an invalid parafoveal preview of the target word. Previews were pronounceable, orthographically legal nonwords, having no positionally overlapping letters with the target, but sharing the same overall shape with respect to ascending, in-line or descending letters (e.g., the preview for *peas* was *gron*). The invalid preview was displayed until participants’ eyes traversed an invisible boundary (located at the end of the pretarget word), when it was replaced by the target which remained on screen until the end of trial.

#### Apparatus and procedure

The apparatus and procedure were identical to Experiment 1 with the exception of the boundary paradigm implementation. Display changes (from preview to target) were made within 6.67 ms (one refresh cycle of the 150 Hz CRT). After the experiment was complete, participants were asked whether they had noticed anything unusual while they were reading. Although many reported seeing ‘something flicker’ on some of the trials, none were able to identify what they had seen. On average, participants answered 97% of comprehension questions correctly.

### Results

Procedures prior to statistical analysis were identical to those in Experiment 1, with the additional elimination of trials when the display change occurred inappropriately. In some cases, the boundary was triggered by a momentary intrusion into the target region as a result of dynamic overshoot of the saccade (e.g., Becker, 1989) which eventually terminated on the pretarget word. In other cases, the boundary was triggered during the target word fixation (due to fixation drift or calibration error). Overall, 16% of the data were excluded. The percentages of remaining data for single fixation, immediate refixation and first-pass skipping of the target were 73%, 14% and 13%, respectively. All analyses of Sent1 data yielded nonsignificant effects (all *F*s < 1) and will not be discussed further. Average values across all measures (with standard deviations) are presented in Table 4, and SFD means across conditions are displayed in Figure 1. For all measures, 3 (Predictability: LP, MP, HP) × 2 (Frequency: LF, HF) ANOVAs (*F*_1_ and *F*_2_) were conducted and are reported in Table 7.

**Table 7.** Main effects and interactions by participants (*F*_1_) and by items (*F*_2_) across measures in Experiment 2.

	Predictability			Frequency			Predictability × Frequency		
	*F*	*MSE*	*p*	*F*	*MSE*	*p*	*F*	*MSE*	*p*
FFD									
*F*_1_	13.77	304	<0.001	17.80	271	<0.001	<1		
*F*_2_	11.95	220	<0.001	12.33	218	<0.01	1.34	212	>0.25
SFD									
*F*_1_	16.54	270	<0.001	26.24	413	<0.001	<1		
*F*_2_	9.82	281	<0.001	27.82	238	<0.001	<1		
GD									
*F*_1_	6.53	765	<0.01	33.90	616	<0.001	1.34	668	>0.25
*F*_2_	4.49	643	<0.05	24.62	422	<0.001	<1		
TT									
*F*_1_	4.96	2327	<0.01	18.07	1558	<0.001	<1		
*F*_2_	2.51	2096	0.092	7.72	1385	<0.05	<1		
PrF									
*F*_1_	3.59	0.004	<0.05	2.15	0.004	>0.15	6.10	0.005	<0.01
*F*_2_	1.91	0.006	>0.15	<1			2.49	0.007	0.098

MSE, mean squared error; FFD, first fixation duration; SFD, single fixation duration; GD, gaze duration; TT, total fixation time; PrF, probability of fixation. Degrees of freedom are as follows: *F*_1_(2, 78) and *F*_2_(2, 48) for Predictability; *F*_1_(1, 39) and *F*_2_(1, 24) for Frequency; and *F*_1_(2, 78) and *F*_2_(2, 48) for Predictability × Frequency.

The main effects of Predictability and Frequency were significant across all measures with the following exceptions: for TT, Predictability was marginal by items; for PrF, Predictability was not significant by items and Frequency was wholly nonsignificant (see Table 7). Follow-up comparisons of Predictability effects are shown in Table 8. Across all measures, there were no differences between LP and MP targets. However, HP targets were, in general, processed faster and fixated less often than both LP and MP targets (see Table 8). Exceptions include the following: LP versus HP was marginal by items in PrF; MP versus HP was marginal by items in GD, marginal by participants and not significant by items in TT, and not significant by participants or items in PrF. For Frequency, fixation times on LF words were longer than those on HF words (FFD: 247 vs 239 ms, SFD: 255 vs 242 ms, GD: 287 vs 268 ms and TT: 328 vs 306 ms, respectively). Finally, there was no evidence of a Predictability × Frequency interaction in fixation times, but there was partial evidence in the PrF measure (see Table 7). This was mainly driven by a Frequency difference in MP words that did not appear in LP or HP words (see Table 4).

**Table 8.** Predictability contrasts by participants (*p*_1_) and by items (*p*_2_) across measures in Experiment 2.

	Mean			LP vs MP		LP vs HP		MP vs HP	
	LP	MP	HP	*p*_1_	*p*_2_	*p*_1_	*p*_2_	*p*_1_	*p*_2_
FFD	247	247	235	>0.95	>0.65	<0.001	<0.001	<0.001	<0.001
SFD	253	253	240	>0.80	>0.95	<0.001	<0.001	<0.001	<0.001
GD	284	279	269	>0.25	>0.30	<0.001	<0.01	<0.05	0.057
TT	328	319	304	>0.20	>0.50	<0.01	<0.05	0.054	0.137
PrF	0.86	0.87	0.88	>0.15	>0.20	<0.01	0.060	>0.20	>0.50

LP, low predictability; MP, medium predictability; HP, high predictability; FFD, first fixation duration; SFD, single fixation duration; GD, gaze duration; TT, total fixation time; PrF, probability of fixation. Mean values are in milliseconds for FFD, SFD, GD and TT.

#### Between-experiment analyses

To explore the effect of Preview (valid vs invalid) and its relationship with Predictability and Frequency, mixed-factor ANOVAs (*F*_1_ and *F*_2_) were performed on the data from Experiments 1 and 2 across all measures. A summary of the main effects of Preview and its interaction with Predictability is provided in Table 9. As before, no effects were found for Sent1 data (all *F*s < 1).

**Table 9.** Preview and Predictability × Preview by participants (*F*_1_) and by items (*F*_2_) across measures.

	Preview			Predictability × Preview		
	*F*	*MSE*	*p*	*F*	*MSE*	*p*
FFD						
*F*_1_	50.89	3510	<0.001	4.88	258	<0.01
*F*_2_	437.02	265	<0.001	3.36	209	<0.05
SFD						
*F*_1_	61.75	3681	<0.001	7.02	243	<0.01
*F*_2_	442.29	336	<0.001	3.81	230	<0.05
GD						
*F*_1_	57.47	8426	<0.001	2.05	528	0.132
*F*_2_	493.26	595	<0.001	1.35	486	>0.25
TT						
*F*_1_	28.75	31,088	<0.001	<1		
*F*_2_	323.38	1635	<0.001	<1		
PrF						
*F*_1_	38.94	0.055	<0.001	7.61	0.006	<0.001
*F*_2_	31.70	0.043	<0.001	5.93	0.006	<0.01

MSE, mean squared error; FFD, first fixation duration; SFD, single fixation duration; GD, gaze duration; TT, total fixation time; PrF, probability of fixation. Degrees of freedom are as follows: *F*_1_(1, 78) and *F*_2_(1, 48) for Preview and *F*_1_(2, 156) and *F*_2_(2, 96) for Predictability × Preview.

Main effects of Preview were found across all measures (see Table 9). Fixation times with invalid previews were longer than those with valid previews (FFD: 243 vs 204 ms, SFD: 249 vs 205 ms, GD: 277 vs 214 ms and TT: 317 vs 231 ms, respectively), and participants were more likely to fixate targets preceded by invalid versus valid previews (0.87 vs 0.74).

A significant Predictability × Preview interaction was observed in early fixation duration measures of FFD and SFD as well as in PrF, but not in GD or TT (see Table 9). As the majority of first-pass fixations were single fixations (94% in Experiment 1, 84% in Experiment 2), only this fixation time measure will be presented (note that statistically identical patterns were found for FFD). Planned follow-up comparisons in SFD revealed that, when Preview was valid, LP targets attracted significantly longer fixations than both MP and HP targets (all *p*s < 0.001) which did not differ from each other (all *p*s > 0.40). When Preview was invalid, however, HP targets attracted significantly shorter fixations than both LP and MP targets (all *p*s < 0.001) which did not differ from each other (all *p*s > 0.95). For PrF, when Preview was valid, a pattern comparable with SFD emerged. LP targets were more likely to be fixated than either MP or HP targets (LP-MP: *p*_1_ = 0.100, *p*_2_ = 0.071; LP-HP: *p*_1_ < 0.01, *p*_2_ < 0.05) which did not differ from each other (all *p*s > 0.90). When Preview was invalid, however, a slightly different pattern emerged. HP targets were somewhat more likely to be fixated than LP targets (*p*_1_ = 0.091, *p*_2_ > 0.20). LP-MP and MP-HP contrasts were not significant (all *p*s > 0.45).

There was no evidence of a Frequency × Preview interaction in fixation time measures (all *F*s < 1). However, for PrF, a significant interaction was found by participants, but it was marginal by items (*F*_1_(1, 78) = 4.30, *p* < 0.05; *F*_2_(1, 48) = 3.82, *p* = 0.057). Follow-up analyses revealed that when Preview was valid, readers were more likely to fixate LF than HF targets (0.76 vs 0.72; all *p*s < 0.001), but when Preview was invalid, the Frequency effect was not reliable (LF = 0.88 vs HF = 0.87; all *p*s > 0.25). Finally, there was no evidence of a Predictability × Frequency × Preview interaction across any measure (for GD, *F*_1_ = 1.45, *p* > 0.20; for PrF, *F*_1_ = 1.85, *p* > 0.15 and *F*_2_ = 1.07, *p* > 0.30; all remaining *F*s < 1).

### Discussion

In the absence of valid Preview, reliable Predictability and Frequency effects were nonetheless observed in fixation duration measures. For Predictability, in general, fixation times on HP words were faster than those on either LP or MP targets which did not differ from each other. For PrF, only Predictability was reliable (by participants) – HP targets were skipped more often than LP items; neither LP versus MP or MP versus HP comparisons reached significance. Unlike Experiment 1, there was no evidence of an interaction between Predictability and Frequency in early fixation measures. Partial evidence of a Predictability × Frequency interaction, however, was found in the PrF measure.

A between-experiments comparison revealed that the presence of invalid parafoveal previews led to increased processing on the target word, evidenced in longer fixation durations and higher fixation probabilities, replicating prior findings (e.g., Blanchard et al., 1989; Sereno & Rayner, 2000a). Critically, however, the Predictability effect depended on Preview for early fixation time measures as well as PrF. Specifically, for FFD and SFD, when Preview was valid, HP as well as MP targets were facilitated relative to LP targets; when Preview was invalid, only HP targets were facilitated (MP and LP targets did not differ). For PrF, a similar but less robust pattern emerged. Preview-dependent effects of Frequency (marginal by items) showed a higher PrF for LF than HF words only when Preview was valid. Taken together, the pattern of findings supports an early parafoveal locus of effects related both to target word predictability and, to a lesser extent, word frequency.

## General discussion

Two experiments tested the effects of Predictability, Frequency and Preview on eye movements during reading. A large, tightly controlled set of materials was devised to address concerns regarding previous investigations in this area. We noted that words labelled as ‘HP’ in past studies were more appropriately classified as MP with respect to the full range of Cloze values. We also suggested that lengthier, individual contextual frames for each target word may prove more effective in terms of their biasing potency. Finally, we employed invalid previews that denied accurate acquisition of parafoveal information but were potentially less disruptive to parafoveal vision than ones that have been used in past studies.

In contrast to previous findings (e.g., Hand et al., 2010, global analyses; Rayner, Ashby, et al., 2004), we found an interaction between Predictability and Frequency in early fixation duration measures when a valid Preview was available. Specifically, under conditions of genuine HP, word frequency effects disappeared. However, no evidence of such an interaction was present when Preview was invalid, although independent effects of Predictability and Frequency were obtained. Nonetheless, Predictability did interact with Preview in early fixation time measures. Valid Preview conferred a relative advantage to both HP and MP targets, whereas invalid Preview impeded LP and MP words. Thus, for MP targets, obtaining a valid parafoveal preview of the target enabled intermediate levels of context to facilitate lexical processing. The pattern of these findings indicates that Predictability plays an early role in the selection of candidate words as readers process a developing discourse. As such, our findings lend support to interactive rather than modular accounts of lexical access. There is growing evidence for a rapid neural sensitivity and response to expectation in visual perception, in particular, via an early top-down influence from the orbitofrontal cortex (i.e., ‘predictive coding’; for example, Trapp & Bar, 2015). It is possible that our use of contexts that were essentially fully predictive of upcoming targets was able to reveal the potency of such facilitatory effects.

Current debates in models of reading are focussed on whether the underlying attentional and cognitive processes involved, in particular at the level of word identification, are serial or parallel in nature. The E-Z Reader model (e.g., Reichle, Rayner, & Pollatsek, 2003) posits that attention is allocated sequentially, word-by-word. In addition, the model assumes a two-stage process of word identification, namely, an initial ‘familiarity check’ (which drives the oculomotor system to program the next saccade) followed by ‘lexical access’ (which signals an attentional spotlight to shift to the next word). In contrast, the saccade-generation with inhibition by foveal targets (SWIFT) model (e.g., Engbert, Nuthmann, Richter, & Kliegl, 2005) holds that attention is allocated as a gradient across several words in parallel and that processing is spatially distributed in relation to retinal eccentricity. In terms of whether Predictability–Frequency effects are additive or interactive, E-Z Reader originally adopted a multiplicative interaction of predictability and frequency (Reichle et al., 2003). However, this function was subsequently revised (Rayner, Ashby, et al., 2004; Reichle, Rayner, & Pollatsek, 2012). In Reichle et al. (2012), predictability can influence processing in two ways. First, in addition to the effects of length and viewing location, target fixation duration can be reduced based on the additive contributions of the target’s predictability and frequency. Alternatively, in some circumstances, context can enable the reader to ‘guess’ the target, resulting in a target word skip. In SWIFT, because predictability is independent of visual input, it can occur earlier than frequency, producing neither a strictly additive nor multiplicative interaction. Thus, in terms of modular and interactive accounts of lexical processing, neither model of eye movement control can be characterized in such simplistic terms. Our data demonstrate both multiplicative and additive patterns of Predictability–Frequency effects that are dependent, critically, not only on the use of genuinely HP contexts but also on whether Preview is valid or invalid. The pattern of Predictability–Frequency findings in Hand et al. (2010) was also dependent on the degree of parafoveal preview (indexed by launch distance). They suggested that oculomotor reading models could implement a preview function (along with fixation duration limits) to generate simulated data that might replicate their findings. The current study provides another dataset with a fuller range of predictability values to validate such models. Systematic implementations of extreme levels of predictability (or anomaly) in reading studies may necessitate a reconsideration of models that simulate performance via neurally plausible mechanisms.

It is important to note that our findings are generally consistent with past eye movement studies. For example, the pattern of Predictability effects in our HF words with valid Preview (LP > MP = HP) replicates that of Rayner and Well (1996) whose targets were HF words. The additive pattern of our Predictability and Frequency effects with valid Preview – when only LP and MP levels are considered – replicates the findings of Rayner, Ashby, et al. (2004) and Hand et al. (2010) whose ‘HP’ targets were more comparable with our MP ones. Finally, the fact that we find interactions of Preview with both Predictability (FFD, SFD, PrF) and Frequency (PrF) substantiates studies showing that Predictability and Frequency effects are modulated by Preview (e.g., Balota et al., 1985; Drieghe et al., 2005; Inhoff & Rayner, 1986; Reingold et al., 2012).

In sum, our study investigated Predictability and Frequency effects on target words in reading. Parafoveal viewing of targets was either maintained or restricted. In normal reading, early fixation measures revealed a Predictability–Frequency interaction. In addition, the nature of the Predictability effect in early fixation measures was contingent on the level of parafoveal preview that had been obtained. The overall pattern of our findings supports an early temporal locus of contextual influence in reading.

## Notes

1. Although Slattery, Staub, and Rayner (2012) disagreed with using a categorical approach in analysis, they did not dispute Hand et al’s (2010) idea of employing launch distance as a metric of parafoveal preview. They suggested that launch distance should be analyzed as a continuous variable (character-by-character) with linear mixed-effect models. Slattery et al. reanalyzed data from two prior eye movement studies that had varied predictability and frequency (Gollan et al., 2011; Staub, 2011), but they failed to replicate Hand et al.’s findings. There are certain concerns, however, with the two studies selected. For example, half of Gollan et al.’s (2011) data were generated by Spanish-English bilinguals whose English vocabulary scores were significantly lower than a control, monolingual English group and who showed greater effects of predictability that were unaffected by frequency. In Staub (2011), participants were presented with each target word (of lower or higher frequency) twice (in lower and higher predictable contexts). It is well-documented that repetition priming confers greater benefit to LF than to HF words (e.g., Forster & Davis, 1984). As such, target repetition reduces the likelihood of finding a Predictability–Frequency interaction. For these reasons, neither dataset reanalyzed by Slattery et al. is optimally placed to serve as comparable replication of Hand et al.

2. The link between pretarget word length and the degree of target parafoveal preview benefit is as follows. Pretarget words that are shorter tend to be higher in frequency (e.g., function words) in comparison with longer, less frequent, content words. It has been demonstrated that the amount of parafoveal information acquired from an upcoming target depends on the difficulty of current foveal processing, with greater preview benefits of a target when the pretarget word is easier to process (e.g., Henderson & Ferreira, 1990). Eye movement behavior, however, is not so straightforward. That is, short, function words are often skipped, leading to greater launch distances to the target (functionally less preview), while longer, content words may receive two consecutive fixations, with a second fixation (having relatively low cognitive load; see Sereno, 1992) typically positioned closer to the target (functionally more preview).

3. Over the years, different types of invalid previews have been used in eyetracking studies: strings of Xs (e.g., Inhoff & Rayner, 1986), visually different words (e.g., Rayner, 1975), random letter strings (e.g., Sereno & Rayner, 1992), nonwords created from position-based single-letter probabilities of words (e.g., Sereno & Rayner, 2000a) and pronounceable nonwords (e.g., Reingold, et al., 2012). Hand et al. (2010) noted that although invalid previews are designed to prevent parafoveal processing of the target, they introduce an incorrect stimulus which can be disruptive. For example, although Sereno and Rayner (2000a) used invalid previews that were relatively word-like, they demonstrated differential effects in target word processing based on their pronounceability. More recently, preview displays have been implemented which present a valid target, but in a form which is either visually degraded (e.g., Gagl, Hawelka, Richlan, Schuster, & Hutzler, 2014) or enhanced (Miellet, et al., 2009).
